# Transposon vector-mediated stable gene transfer for the accelerated establishment of recombinant mammalian cell pools allowing for high-yield production of biologics

**DOI:** 10.1007/s10529-020-02889-y

**Published:** 2020-04-22

**Authors:** Natalie Tschorn, Karen Berg, Jörn Stitz

**Affiliations:** 1 Research Group Pharmaceutical Biotechnology, TH Köln – University of Applied Sciences, Chempark Leverkusen E28, Kaiser-Wilhelm-Allee, 51368 Leverkusen, Germany; 2 Institute of Technical Chemistry, Leibniz University Hannover, Hannover, Germany; 3 Research Group Translational Hepatology and Stem Cell Biology, Cluster of Excellence REBIRTH, Department of Gastroenterology, Hepatology, and Endocrinology, Hannover Medical School, Hannover, Germany

**Keywords:** Mammalian cells, *piggyBac*, Protein production, *Sleeping Beauty*, Transposon vector

## Abstract

Stable recombinant mammalian cells are of growing importance in pharmaceutical biotechnology production scenarios for biologics such as monoclonal antibodies, growth and blood factors, cytokines and subunit vaccines. However, the establishment of recombinant producer cells using classical stable transfection of plasmid DNA is hampered by low stable gene transfer efficiencies. Consequently, subsequent selection of transgenic cells and the screening of clonal cell populations are time- and thus cost-intensive. To overcome these limitations, expression cassettes were embedded into transposon-derived donor vectors. Upon the co-transfection with transposase-encoding constructs, elevated vector copy numbers stably integrated into the genomes of the host cells are readily achieved facilitating under stringent selection pressure the establishment of cell pools characterized by sustained and high-yield recombinant protein production. Here, we discuss some aspects of transposon vector technologies, which render these vectors promising candidates for their further utilization in the production of biologics.

## Introduction

With the growing demand for biotherapeutics at highest quality standards, mammalian cells are increasingly used for production. In 2018, 84% of the marketed biotechnologically produced drugs were generated using mammalian cells. The vast majority of these biologics are therapeutic monoclonal antibodies produced in Chinese hamster ovary (CHO) cells (Walsh 2018).

Recombinant mammalian protein producer cell lines are not only required for the final industrial scale production of proteins of interest (POIs). They are already needed in early phases of drug discovery and development. POIs are produced at research laboratory-scale to facilitate their biochemical and biophysical characterization followed by preclinical trials in small animal models in vivo. The classical approach to establish recombinant cell lines uses circular plasmid DNA for stable transfection (Manceur et al. 2017). Although a wide range of transfection methods and reagents are available, some host cells show low transfectability. Even if the transient transfection efficiency—i.e. the epichromosomal cellular uptake of plasmids—is sufficient, the stable integration of linearized plasmid DNA into the host cell genome is a rare event. Depending on the host cell type, the donor organism and the transfection protocol used, most stably transfected cells only harbor low vector copy numbers (VCN) ranging from single to about five plasmid molecules randomly integrated into the host cell genome (Chusainow et al. 2009; Kolacsek et al. 2014). The integrated plasmid sequences do not only contain the expression cassette entailing a promoter/enhancer required to drive the expression of the gene of interest (GOI) encoding the POI and a polyadenylation signal (p(A)), but also long bacteria-derived sequences. At least an origin of replication (*ori*) and an antibiotic-resistance gene allowing for the selection and subsequent amplification of the plasmid in transformed bacteria are required. These bacterial sequences are recognized as foreign and targeted by the methylation machinery in mammalian cells. This results in silencing of proximate eukaryotic or viral promoter/enhancer driving the expression of the GOI (Riu et al. 2007) and can consequently prevent sustained high-level POI production. Typically, polyclonal or pooled cell populations do not produce satisfying POI yields. Thus, the screening of hundreds or even thousands of cell clones to identify a protein producer cell demonstrating efficient, stable and sustained expression levels of POI is indispensable (Wang et al. 2018). In summary, using stable transfection, all aforementioned limitations contribute to the time-consuming and cost-intensive processes to establish protein producer cells. Novel vector technologies are required to accelerate the generation of transgenic cells at early drug development stages. Vector systems derived from transposons were developed and further improved in the past 15 years allowing the rapid establishment of stable recombinant mammalian cell pools for the high-yield production of biologics.

## DNA transposon biology and host cell interaction

DNA transposons are fossil mobile DNA elements. A wide range of individual DNA transposons from different families and donor organisms were characterized in detail over the last three decades e.g. *Tol2* originating from medeka fish, *Sleeping Beauty -* synthetic sequences derived from transposons found in the white cloud minnow, atlantic salmon and rainbow trout—and *piggyBac* isolated from the cabbage looper moth (Fraser et al. 1996; Ivics et al. 1997; Kawakami et al. 1998). All DNA transposons are composed of a transposase gene and flanking inverted terminal repeats (ITRs; Muñoz-López and García-Pérez 2010). The enzyme transposase recognizes specific short target sequences, called directed repeats (DRs) located in the ITRs. Upon binding, the transposase cuts out the transposon sequence from the surrounding genomic DNA of the host cell. The formed complex consisting of the mobilized transposon DNA fragment and the still bound transposases is now able to change its position to a new location in the cell genome. The transposases open the genomic DNA backbone at the new *locus* and insert the transposon fragment. The ligation of the open DNA ends is mediated by cellular key factors of the non-homologous end joining pathway (NHEJ) within the double strand break (DSB) repair system (Mátés et al. 2007). Thus, this so called transposition uses a cut-and-paste mechanism.

The examination of the sequences targeted by the respective transposases for re-integration into the genomic DNA of the host cell revealed differences between various transposons. While *Tol2* of the *hAT* family could not be shown to prefer a specific sequence, members of the *Tc1/mariner* family like *Sleeping Beauty* (SB), *Frog Prince* and *Minos* as well as *piggyBac* (PB; superfamily PB) clearly favor defined insertion motifs. With the dinucleotide TA for *Tc1/mariner* transposons and the four-nucleotide motif TTAA for PB, these target sequences are very short, and thus would allow close- to-random integration over the entire host cell genome (Grabundzija et al. 2010). This assumption was further supported by the findings that *Tol2*, SB and PB did not show any preference for specific host cell chromosomes. *Tc1/mariner* transposons including SB were demonstrated to perform close-to-random integration. Although not very pronounced, there seems to be a weak bias in mammalian cells towards the insertion into transcribed regions and their regulatory sequences located upstream (Yant et al. 2005; Huang et al. 2010; Gogol-Döring et al. 2016). In contrast, *Tol2* and PB favor certain specific genomic regions. Both, *Tol2* and PB, insert mostly upstream and in close proximity to transcriptional start sites (TSSs), CpG-islands and DNase I hypersensitive sites (Huang et al. 2010). For PB it was recently shown (Gogol-Döring et al. 2016) that the cellular BET proteins interact with the transposase and guide the accumulation of insertions to TSSs. In this regard, PB shows a high similarity to the γ-retrovirus murine leukemia virus (MLV; Wu et al. 2003; de Jong et al. 2014; Gogol-Döring et al. 2016).

Only a few cellular proteins interacting with the transposase have been described to date. In a yeast two-hybrid screen the transcription factor Myc-interacting protein zinc finger 1 (Miz1) was identified to interact with SB transposase (Walisko et al. 2006). As a result the expression of cyclin D is down-regulated in transgenic human cells leading to a temporary arrest in cell cycle phase G_1_. Integration into the host cell genome appears to be more efficient during a prolonged G_1_ phase. The DNA-bending high mobility group protein 1 (HMGB1) was shown to be crucial to facilitate efficient transposition. While transposition was largely limited in HMGB1-deficient murine cells, this restriction was abrogated by transient recombinant over-expression of HMGB1 and partially overcome by HMGB2. It is assumed, that at least HGMB1 serves as a co-factor for binding of the transposase to the target DR sequences in the ITRs, and thus supporting the formation of the synaptic transposase-DNA complex during transposition (Zayed et al. 2003). In contrast, transposition of PB appears to be largely cell factor independent as it can be experimentally reconstituted in vitro using purified PB transposase and DNA elements (Burnight et al. 2012). Like retroviruses, SB as well as PB seem to exploit the cellular barrier to autointegration factor (BAF) to promote transposon integration into the host genome at high efficiencies by preventing autointegration (Wang et al. 2014).

**Fig. 1:**
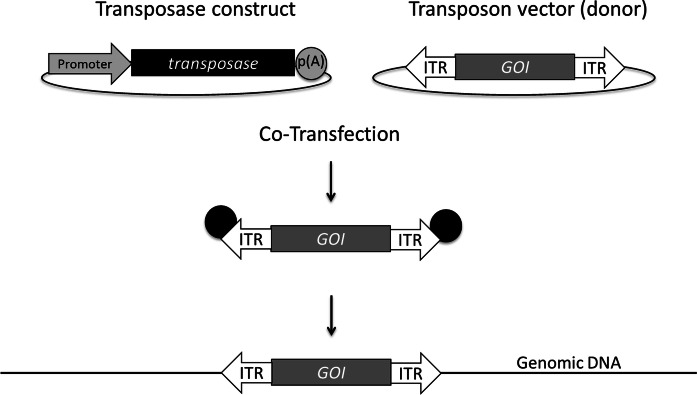
Two-component transposon vector systems consist of a transposase construct minimally encompassing a promoter/enhancer upstream of the transposase coding region and a polyadenylation signal (p(A)). The transposon or donor vector contains the gene of interest (GOI) flanked by the inverted terminal repeats (ITRs). Upon co-transfection into suitable host cells, the transposase expressed in trans binds to the ITRs and cuts out the transposon vector fragment from the bacterial plasmid backbone and mediates the integration into the cell genome using a cut-and-paste mechanism

**Fig. 2:**
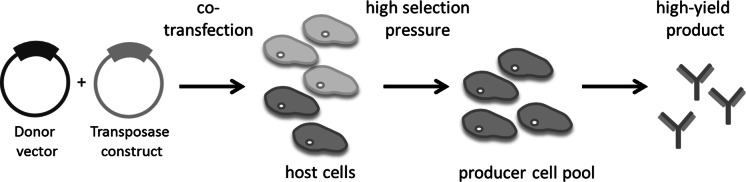
Rapid establishment of protein producer cells using a two-component transposon vector system by co-transfection of the donor vector and the transposase construct into host cells. One or two days post transfection, selection pressure can be applied to generate a high-yield producer cell pool

### DNA transposon vectors

As illustrated in Fig. 1, in a two-component DNA transposon-derived vector system the transposase gene and the ITRs are separated onto two different plasmids. The transposase construct minimally entails a suitable promoter active in the desired host cell, the sequence encoding the transposase and a 3’-located p(A). The donor or transposon vector encompasses an expression cassette with the GOI flanked by the ITRs. Upon co-transfection of target cells with both plasmids, the transposase is expressed and cuts out the GOI expression cassette framed by the ITRs from the plasmid backbone. The subsequent stable insertion of the transposon vector into the genome concludes the transposition process (Ivics et al. 1997). In contrast to transgenic cells established by classical stable transfection using plasmids, the integrated transposon vectors do not contain any bacterial sequences originating from the plasmid vector backbone. Consequently, silencing of the promoter driving GOI expression should presumably occur at much lower frequency (Riu et al. 2007). Amongst other features, this renders transposon vectors attractive tools for the establishment of recombinant protein producer cells.

Vector systems derived from SB and PB are most frequently used for biotechnological applications. However, the first generation of transposon vectors derived from SB was characterized by low transposition efficiencies (Ivics et al. 1997). The stepwise optimization of the SB transposase by the introduction of accumulating mutations led in a pioneering study to the evolved enzyme SB100X revealing a 100-fold improved activity as compared to the wild-type protein (Mátés et al. 2009; Voigt et al. 2016). The transposase of PB, namely mPB, was first adapted to mediate 20-fold enhanced transposition efficiency in mammalian cells employing codon optimization (Cadiñanos and Bradley 2007; Liang et al. 2009). Following the example of improving transposase activity by means of molecular evolution (Mátés et al. 2009), the mPB enzyme was further advanced. The resultant hyPB was demonstrated to mediate 10-fold enhanced transposition activity in mammalian cells as compared to mPB (Yusa et al. 2011). Cui and co-workers (Cui et al. 2002) described the optimization of the ITR sequences of SB transposon vectors leading to a four-fold increase of transposition events in human HeLa cells. Later attempts to improve transposition efficiencies of SB vectors by the introduction of additional point mutations within the ITRs did unfortunately not lead to further improvements (Scheuermann et al. 2019).

The achieved transgenic rate—i.e. the percentage of stably genetically modified cells as a result of stable integration of the donor vector into the host cell genome—using SB and PB two-component vector systems obviously depends on the transfectability of the desired target cell and the transfection protocol employed, which may require individual optimization. The efficiency of transposition can be divided into three different subsequent events: (i) the excision of the transposon vector from the plasmid backbone, (ii) the overall stable transgenic rate and (iii) the average VCN per cell (Kolacsek et al. 2014). The transposition efficiencies observed in different cell types are highly variable (Izsvák et al. 2000; Troyanovsky et al. 2016). Besides the amount of plasmid DNA transfected per target cell count (high versus low dose), optimization of the transposition rate and VCN per cell can be improved by determining the optimal ratio of transposase construct to transposon vector (Grabundzija et al. 2010). For the utilization of SB-derived vector systems, it has to be emphasized that an excess of transposase hampers efficient transposition—a phenomenon termed overexpression inhibition (OPI). It was hypothesized that a high concentration of transposase saturates the target DR sequences in the ITRs slowing synapsis formation of transposon ends (Liu and Chalmers 2014). Consequently, the optimal SB transposase construct to donor ratio, depending on the genetic design of the expression cassettes, ranges in most cell types from 1/5 to 1/30. PB transposase does not suffer from OPI (Wilson et al. 2007). Nevertheless, also for PB systems the optimal balance of vector components has to be examined for every target cell aiming to achieve the highest transgenic rate.

Higher transgenic rates are generally achieved using larger amounts of transposon vector molecules in high dose transfection protocols (e.g. >500 ng DNA per 1 × 10^5^ cells). Depending on the host cell line, its transfectability and its assistance in transposition, about 10 to 30% of the transfected cells are stably modified. The DNA dosage also influences the integrated copy numbers per cell. Using low dose approaches (15–50 ng per 1 × 10^5^ cells) the vast majority of transgenic cells show single vector integrations. Here, usually 1 to 10% of the transfected cells reveal stable vector integration. In contrast and upon high dose transfection, PB transposon vectors reach single to single-digit number of vector insertions per cell, respectively. However, SB100X facilitates up to 40 integrations under these conditions (Grabundzija et al. 2010). The high vector copy numbers per cell make SB vectors attractive for the establishment of stable producer cell lines. However, PB vectors seem to have the advantage of targeting favorable sites close to TSSs resulting in higher levels of transgene expression. Besides the high transposition rates achieved with both transposon vector systems, SB and PB qualify for a wide range of biotechnological applications due to their considerable high cargo capacity. SB vectors were reported to be instrumental in gene transduction carrying payloads of up to 6 kb. Larger transgene cassettes lead to declining transposition frequencies (Izsvák et al. 2000; Rostovskaya et al. 2012). However, SB and PB vectors were demonstrated to mediate stable transgene transfer of even larger DNA molecules such as artificial bacterial chromosomes (BACs) with sizes of up to impressive 200 kb outperforming retroviral vectors with maximum cargo capacities of about 6 kb (Li et al. 2011; Rostovskaya et al. 2012).

### Transposon vectors for the accelerated establishment of mammalian protein producer cell pools

Due to their ability to stably transduce genetic cargo into a variety of mammalian cell types, transposon vectors, particularly derived from SB and PB, provide a toolbox for many applications. These include the generation of transgenic animals, the development of gene knock-out screens in gene function discovery, the establishment of inducible pluripotent stem cells (iPSCs) and the utilization in somatic gene therapy (Di Matteo et al. 2012; Yusa 2015; Narayanavari et al. 2017; Tipanee et al. 2017). In these fields, transposon vectors are increasingly used as alternatives to retroviral vector-mediated gene transfer (Vargas et al. 2016).

The generation of polyclonal pools of genetically modified mammalian cells for the purpose of recombinant protein production was first demonstrated using MLV and HIV-1 vectors mediating gene transduction (Oberbek et al. 2011; Stitz 2011; Elegheert et al. 2018). These approaches capitalized on the stable integration of multiple vector copies per cell and the sustained expression of the transgenes as a result of the favored insertion sites located in the proximity of transcriptional start sites and active cellular transcription units (Craigie and Bushman 2014; Gogol-Döring et al. 2016). The productivity of the recombinant cell pools and cell clones established upon viral vector-mediated gene transduction was remarkable. However, the utilization of viral vector technology requires (i) the establishment of stable or transient viral packaging cells, upon co-expression of at least three different constructs, namely, the transfer vector harboring the transgene of choice, a packaging construct minimally encompassing the structural genes *gag* and *pol* encoding for the viral core proteins and enzymes and an envelope construct enabling the expression of the Env proteins facilitating vector particle cell entry. (ii) The subsequent titration of produced vector particle preparations in suitable susceptible target cells has to be conducted. (iii) In case vector particles with a tropism for human cells or pantropic vectors pseudotyped with the G-protein of vesicular stomatitis virus (VSV-G) are used, a biosafety level 2 laboratory (BSL-2) is indispensable. In summary, the complexity of viral vector production and characterization reduces the attractiveness of retrovirus technology for the establishment of transgenic cell pools for protein production.

The utilization of transposon vectors is comparably convenient and straightforward as illustrated in Fig. 2. The required optimization of transfection protocols and the assessment of the optimal transposase construct to transposon vector ratio for the host cell of choice are standard practices easily established in any laboratory and are quickly performed by experienced staff. Usually, the generated recombinant mammalian cells are classified as genetically modified organisms of BSL-1, provided that the mobilized payload is not hazardous itself e.g. oncogenes. PB and SB vector systems are both instrumental in the rapid establishment of transgenic cells in vitro. Multiple stable insertions of vectors in the host cell genome are readily achieved. With a possible payload of far more than 6 kb, both transposon vector systems exceed the capacity requirements for the purpose of protein production. Even two protein encoding expression cassettes *in cis* are easily mobilized, e.g. for the co-production of heavy and light chains of monoclonal antibodies (mAbs; Ahmadi et al. 2017).

Matasci et al. (2011) were the first to use PB vectors to establish pools of CHO cells producing tumor necrosis factor receptor (TNFR) fused to the Fc-fragment of human immunoglobulin G1 (IgG1). The bicistronic donor vector entailed a cytomegalovirus promoter/enhancer (P_CMV_) driving the expression of the fusion protein and a herpes simplex virus thymidine kinase promoter (P_HSV-TK_) coupled to a *pac* gene mediating resistance against puromycin. Using a mPB transposase expression construct to donor vector ratio of 1:9, transgenic cells were established upon co-transfection. One day post transfection, cells were subjected to selection by expansion in the presence of puromycin at a concentration of 10 and 50 µg/ml. Both resultant stable cell pools—PB(10) and PB(50), indicating the selection pressure applied—facilitated considerable product yields of 42 and 50 mg/l TNFR:Fc, respectively. A number of cell clones were isolated from both pools and analyzed for their productivity. While more than 34% of the PB(50)-derived clones revealed a productivity reaching 50 to 100 mg/l, only 28.6% of the clones originating from the parental PB(10) pool were as productive. This difference in cell productivity was even more visible for yields between 25 and 50 mg/l with 55.8% of all PB(50)-derived clones and only again 28.6% of PB(10) cell clones. Only less than 10% of the PB(50) cell clones produced less than 25 mg/l. In contrast, 40% of PB(10) clones were significantly less productive than the parental pool. As expected, enhanced productivity correlated with higher VCN per cell ranging from an average of three copies for the least productive clones up to six copies for the best performing ones. In conclusion, this demonstrated the rapid establishment of cell pools with advanced productivity employing PB vectors and very stringent selection. Noteworthy, cell productivity of pools and clones was stable over a period of three months in the absence of selection (Matasci et al. 2011).

In 2016, the research team led by Dr. Wurm used the above described expression cassettes in transposon vectors derived from *Tol2*, SB and again PB to compare the three vector systems for their potential to establish CHO producer cell pools and clones (Balasubramanian et al. 2016a). The optimal donor to transposase construct ratio for each vector system using mTol2, mPB and SB100X transposases was assessed. *Tol2* vectors showed dramatically lower transposition efficiencies as compared to SB and PB. In fact, even with the best performing donor to transposase construct ratio employed, at least over 60% of the inserted *Tol2* vectors were demonstrated to be a result of stable transfection rather than transposition. Consequently, and in the absence of selection pressure, cell pools established with PB and SB vectors generated significantly higher transgenic rates of about 20% stably modified cell populations as compared to less than 5% using the *Tol2*-derived system. In addition, SB and PB vectors reached more than three-fold higher transgene expression levels than *Tol2* vectors. However, and upon application of a low selection pressure (10 µg puromycin/ml) for ten days, pools of cells were established reaching productivities ranging from 80 mg/l of TNFR:Fc using *Tol2* vectors to 95 mg/l for PB and SB vectors. On average, nine-fold higher yields were obtained using transposase-mediated gene transfer as compared to conventional stable transfection of plasmids. In 14-day fed-batch cultures of pools, TNFR:Fc levels of up to 900 mg/l were achieved. Over a period of three months in the absence of selection pressure, productivity decreased to approximately 50% of the initial yields for all pools generated with the three vector systems. This underscored the necessity of stringent selection to establish sustained high-yield expression.

PB vectors were also utilized to establish CHO cell pools for the production of monoclonal antibodies. The expression of heavy and light chain was driven by a P_CMV_ whereas a SV40 promoter mediated the expression of glutamine synthetase (GS). These expression cassettes present on one donor vector were flanked by insulator sequences to minimize post-transpositional silencing. Stable recombinant cell pools cultivated in shaker flasks were established producing four different mAbs. Yields of 2.3 to up to remarkable 7.6 g/l were reported (Rajendra et al. 2016). Unfortunately, the identity of the produced antibodies was not disclosed. The high productivities of the cell pools were shown to originate from up to seven VCN per cell leading to high expression levels of mRNA. Most importantly, the cell pools revealed a very high homogeneity resulting from highly productive clones composing the vast majority of the population. The scale-up from shaker flasks to a 36 l bioreactor delivered yields of 4.7 g/l for one of the monoclonal antibodies (Rajendra et al. 2017a). CHO pools established using PB vector-mediated gene transduction produced mAbs of the same quality as compared to mAbs harvested from clonal producer cell lines generated by stable transfection. This finding was also confirmed in a second study conducting capillary electrophoresis-sodium dodecyl sulfate (CE-SDS), glycan analysis, analytical size-exclusion chromatography (aSEC) and peptide mapping analysis using liquid chromatography and mass spectrometry (LCMS; Rajendra et al. 2017b).

High yields of multiple products can also be achieved by the generation of cell pools using simultaneous co-expression from multiple individual transposon vectors upon co-transfection with a transposase construct. The team around Balasubramanian (Balasubramanian et al. 2016b) demonstrated the establishment of CHO cell pools producing three model proteins, namely, enhanced green fluorescent protein (EGFP), secreted alkaline phosphatase (SEAP) and a monoclonal antibody. Not surprisingly, the productivity of the pools was superior when each individual transgene encompassing transposon vector carried a different selectable marker allowing for the application of three separate selection pressures at the same time. Accordingly, we recently reported on the rapid establishment of highly efficient viral packaging cell lines (VPCs) using SB-derived transposon vectors in human HT-1080 fibrosarcoma cells (Berg et al. 2019). All three vector components required—the packaging and *env*-construct and the transfer vector as well—were co-transfected with the transposase construct and allowed for subsequent triple-selection. Within only three weeks, VPCs were established reaching vector particle yields of over 1.0 × 10^6^ transducing units per ml (TU/ml). In contrast, VPCs that were generated by conventional stable plasmid transfection achieved 20-fold lower titers and required an extended establishment time of three months.

The previously mentioned findings demonstrate the utility of transposon vectors for the future generation of cell pools to rapidly produce second generation biologics composed of multiple components. Examples for these complex biologics are viral vector particles for somatic gene therapy and enveloped virus-like particle (VLP) vaccines displaying single or multiple target antigens at high density on their surface. There is a demand in the pharmaceutical industry for accelerated producer cell line development without lowering product yields or compromising product quality to maintain the provision of biologics at economically affordable prices to the growing market.

## Conclusions and outlook

Amongst others, transposon vector systems derived from *Sleeping Beauty* and *piggyBac* are most frequently used for biotechnological applications. Transposases were optimized and donor vectors further developed enabling the rapid establishment of highly productive recombinant cell pools. Upon a brief optimization of the co-transfection method and the donor vector to transposase construct ratio, a wide range of host cells is susceptible to these relatively new vector systems. The technology capitalizes on the efficient stable integration of multiple transgene expression cassettes per cell. Using the industry gold standard CHO as host cells, transgenic rates of 20% are readily achieved. Antibiotic resistance marker genes coupled to the transgene of choice facilitate the stringent selection required to establish highly productive pools of cell clones in a short time. Transposon vectors were shown to overcome the limitations of conventional stable plasmid transfection and will thus prove their utility and value for the future development of novel biologics and their industrial scale production.

However, the development of these vectors is still in an early pioneering stage. Most importantly, the vector design can certainly be further improved. Some advancement was already achieved in enhancing expression of transgenes by insertion of matrix attachment regions (MARs; Ley et al. 2013). To minimize gene silencing, the utilization of flanking cHS4 DNA insulators was instrumental (Sharma et al. 2012). Transposon vectors encompassing inducible transgene expression cassettes for the production of cytotoxic proteins were generated (Kowarz et al. 2015; Michael and Nagy 2018). To avoid the use of antibiotics during transgenic cell selection and product manufacturing, vectors were designed for the establishment of cell pools upon chemically induced dimerization of growth factor receptors (Kacherovsky et al. 2012). Novel panels of transposon vectors will have to be tailored combining multiple genetic elements and vector components to further enhance expression levels and to meet specific requirements of future industrial production scenarios.
